# Diffusion of good practices of care and decline of the association with case volume: the example of breast conserving surgery

**DOI:** 10.1186/1472-6963-7-167

**Published:** 2007-10-18

**Authors:** Ugo Fedeli, Natalia Alba, Elena Schievano, Cristiana Visentin, Rosalba Rosato, Manuel Zorzi, Giancarlo Ruscitti, Paolo Spolaore

**Affiliations:** 1 SER-Epidemiological Department, Veneto Region. Via Ospedale 18-31033 Castelfranco Veneto (TV), Italy; 2 Unit of Cancer Epidemiology, S. Giovanni Battista Hospital and University of Turin. Via Santena, 7 – 10126 Torino, Italy; 3 Venetian Tumour Registry, Istituto Oncologico Veneto (IOV). Via Gattamelata, 64 – 35128 Padova, Italy; 4 Department of Health and Social Services, Veneto Region. S. Polo, 2513 – 30125 Venezia, Italy

## Abstract

**Background:**

Several previous studies conducted on cancer registry data and hospital discharge records (HDR) have found an association between hospital volume and the recourse to breast conserving surgery (BCS) for breast cancer. The aim of the current study is to depict concurrent time trends in the recourse to BCS and its association with hospital volume.

**Methods:**

Admissions of breast cancer patients for BCS or mastectomy in the period 2000–2004 were identified from the discharge database of the Veneto Region (Italy). The role of procedural volume (low < 50, medium 50–100, high > 100 breast cancer surgeries/year), and of individual risk factors obtainable from HDR was assessed through a hierarchical log-binomial regression.

**Results:**

Overall, the recourse to BCS was higher in medium (risk ratio = 1.12, 95% confidence interval 1.07–1.18) and high-volume (1.09, 1.03–1.14) compared to low-volume hospitals. The proportion of patients treated in low-volume hospitals dropped from 22% to 12%, with a concurrent increase in the activity of medium-volume providers. The increase over time in breast conservation (globally from 56% to 67%) was steeper in the categories of low- and medium-volume hospitals with respect to high caseload.

**Conclusion:**

The growth in the recourse to BCS was accompanied by a decline of the association with hospital volume; larger centers probably acted as early adopters of breast conservation strategies that subsequently spread to smaller providers.

## Background

Breast conserving surgery (BCS) followed by radiotherapy to the residual breast tissue has been proved as an appropriate approach to the treatment of early-stage breast carcinoma since the early 1980s [1]. Although a consensus on breast conservation has been reached since 1990 in the United States [2], mastectomy rates after the introduction of these recommendations have been higher than expected and unexplained by medical contraindication [3,4].

Large centers are more likely to adopt innovative therapeutic modalities [5], and early studies from both Northern America and Europe showed a significant association between the surgical approach to breast cancer and hospital bed size or procedural volume (yearly number of breast cancer surgeries) [5-8]. More recent investigations conducted on data from cancer registries or hospital discharge records (HDR) continue to detect this pattern. For example, BCS was more frequently performed in high-volume hospitals in Los Angeles County and New York City in the period 1990–1998 [9,10], as well as in an analysis of thirteen years (1988–2000) of the US Nationwide Inpatient Samples [11]. Moreover, the finding of a significant association between the recourse to BCS and hospital volume has been replicated in analyses of HDR from some Italian regions [12-15].

In the Veneto Region located in northeastern Italy, breast cancer is a major public health problem, as the crude rate was 167 per 100,000 individuals in the period 1998–2001, with an estimated 1.96% annual percent increase in incidence from 1987 to 2001 [16]. The aim of the present study is to examine HDR and assess time trends in the recourse to BCS in Veneto, as well as to investigate concomitant changes in the role -if any- played by hospital volume.

## Methods

The total population of the Veneto Region was 4,699,950 on December 31, 2004 (ISTAT-National Institute of Statistics). There were approximately 950,000 discharges from Veneto hospitals each year; one primary and up to 5 secondary discharge diagnoses and one primary and up to 5 secondary procedures were registered in HDR.

All discharges of residents from Veneto hospitals with the International Classification of Diseases, 9^th ^Revision-Clinical Modification (ICD9-CM) code 174 (cancer of the female breast) in all diagnostic categories, were identified for the period January 1, 2000 to December 31, 2004. Among these, admissions with BCS (ICD9-CM procedure codes 85.21–85.23) and mastectomy (85.41–85.48) were selected; patients who had already previous breast surgery in 1999 were excluded. In the case of repeated admissions for breast surgery in the years 2000–2004, only the first admission was considered. Lastly, we also included surgical biopsies (ICD9-CM procedure code 85.12) as BCS when no other breast surgery was performed in the study period, assuming that the biopsy resulted in complete excision of early breast cancer. A parallel analysis was also conducted examining the less conservative surgery identified in 2000–2004 among repeated admissions for breast surgery.

**Figure 1:**
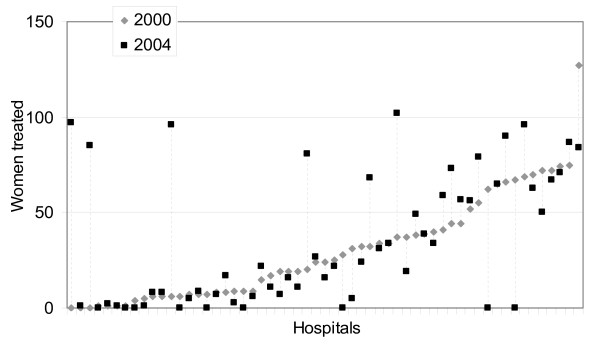
Hospital caseload in 2004 compared to 2000 (seven hospitals with more than 100 surgeries were excluded in both years).

The variables examined were patient's age (four classes), severity of cancer defined by clinical criteria for disease staging adapted to ICD9-CM codes [17] (classified as nonlocalized when discharge codes 196–198 of locoregional or distant metastasis were reported), presence of comorbidities (an adaptation of the Charlson Comorbidity Index computed on the index admission and on admissions in the previous year through a program from the National Cancer Institute [18]), calendar year of discharge, and the hospital's volume of activity (<50, 50–100, >100 women with breast cancer surgery). The volume cut-off points were chosen a priori in order to compare results with recent studies carried out in Northern Italy [14,19]. Since the case volume sharply fluctuated in each hospital through the study period (Figure 1), a hospital's activity was defined as the annual number of discharges with breast surgery and was allowed to vary each year.

A hierarchical log binomial regression was applied in order to assess the influence of the above variables on the recourse to BCS while taking into account clustering of patients within providers. Since BCS is a common event, odds ratios estimated through conventional logistic regression depart from risk ratios (RR) of breast conservation. We therefore applied a model that directly estimates the RR [20]. The analysis was performed through the gllamm procedure of the package Stata 8 by assuming a binomial distribution and a log link function. To investigate time changes in the association of breast conservation with hospital caseload, an additional analysis was conducted including, as well as age, severity and comorbidity, a dichotomized variable for volume of activity (large versus medium and small hospitals), a linear term for the calendar year, and an interaction term between the latter two variables.

## Results

Overall, 18,584 HDR with a diagnosis of female breast cancer had BCS or mastectomy procedure codes in the five years examined. Of the above records, 1,391 repeated admissions (7.5% of the hospitalizations for breast surgery) and 152 patients with breast surgery in 1999 (0.8%) were excluded; it is worth noting that 77.6% of the 1,391 repeated admissions occurred within 90 days from the first surgery. Lastly, 1,603 surgical biopsies with no other surgery recorded through the study period were added to the dataset, resulting in a total of 18,644 records (representing unique patients) submitted to statistical analyses.

The distribution of patients' age was unbalanced between hospitals groups: patients ≥70 years represented 40% of those treated in low-volume hospitals, while constituting only 33% and 25% in medium- and high-volume hospitals, respectively; only minor differences were evident for presence of comorbidities and stage of disease.

The BCS rate was 61.5% overall and markedly lower in those aged 70 or more, in patients with nonlocalized disease, in the presence of one or more comorbidities, and in those treated in low-volume hospitals following regression analysis; the recourse to conservative surgery sharply increased with calendar year and was above 65% at the end of the study period (Table 1).

**Table 1:** Discharges with breast surgery (n), percentage of breast conserving surgery (BCS), Risk Ratios for BCS (RR) with 95% Confidence Intervals (CI) estimated by hierarchical log binomial regression.

	n	BCS (%)	RR	CI
	
*Age *(*yrs*)				
<50	3995	68	ref	
50–59	4260	70	1.04	1.02 – 1.07
60–69	4806	67	1.01	0.98 – 1.04
70+	5583	46	0.72	0.69 – 0.75
*Severity of disease*				
Localized	17145	63	ref	
Non-localized	1499	47	0.73	0.69 – 0.77
*Comorbidity index*				
0	17391	62	ref	
1+	1253	49	0.90	0.85 – 0.96
*Hospital volume*				
<50	3249	52	ref	
50–100	5685	60	1.12	1.07 – 1.18
>100	9710	66	1.09	1.03 – 1.14
*Calendar year*				
2000	3541	56	ref	
2001	3721	59	1.05	1.01 – 1.08
2002	3807	61	1.07	1.03 – 1.11
2003	3858	64	1.13	1.09 – 1.17
2004	3717	67	1.15	1.11 – 1.19

During the five years observed there was a progressive increase of patients discharged from medium-volume hospitals, whereas the proportion treated by structures with less than 50 admissions for breast surgery fell from 22% to 12% (Table 2). In the first year studied, high-volume hospitals showed the highest BCS rate, although, thereafter the increase over time in the proportion of breast conservation was steeper in the categories of low and medium annual caseload; in 2003–2004 the recourse to BCS was similar in medium- and high-volume structures (Table 2). Similar results were obtained when the less conservative instead of the first surgery was assigned to patients with repeated admissions (data not shown).

**Table 2:** Annual proportion of women treated by low (<50 surgeries), medium (50–100), and high volume hospitals (>100), and corresponding percentage of breast conservation (BCS%)

	Proportion of women treated			BCS%		
		
	<50	50–100	>100	<50	50–100	>100
		
2000	0.22	0.23	0.55	47	50	63
2001	0.21	0.30	0.49	46	61	65
2002	0.17	0.32	0.51	54	57	66
2003	0.16	0.29	0.56	58	65	66
2004	0.12	0.38	0.50	58	66	69

**Figure 2:**
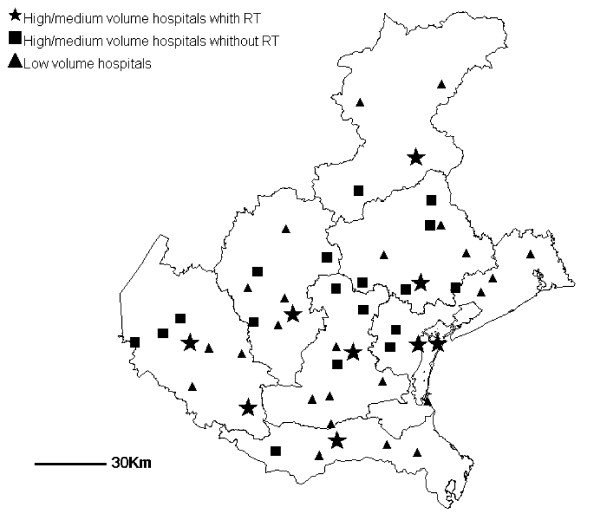
Veneto Region, 2004: geographical distribution of centers performing breast cancer surgery by presence of a radiation therapy facility (RT) and case volume (low < 50, medium/high ≥50).

The decrease in the percentage of patients treated in low-volume hospitals is partially explained by ward closures or amalgamations (Table 3): of 41 low-volume hospitals in 2000, 8 no longer were performing breast surgeries by the end of the study period, 7 increased their activity above 50 surgeries, 26 remained under 50 surgeries (and overall reduced their caseload); an additional low-volume center was active in 2004. It is worth noting that among the nine radiation treatment facilities available in the Veneto Region, four were placed in medium-volume and five in high-volume hospitals; two of the latter were academic centers. The geographical distribution of centers by case-volume and availability of adjuvant radiation therapy is shown in Figure 2.

**Table 3:** Number of centers performing breast cancer surgery at the beginning and the end of the study period, and presence of a radiation therapy capability according to hospital volume

Hospital volume	n centers		availability of radiation therapy (2004)
	2000	2004	
		
<50	41	27	0
50–100	12	19	4
>100	8	8	5

The age-severity-comorbidity adjusted RR for BCS (with 95% Confidence Interval) was 1.12 (1.09–1.14) for large versus small and medium institutions, 1.06 (1.05–1.07) for the linear trend through calendar years, and 0.98 (0.97–0.99) for the interaction term. This latter result confirms that the influence of hospital caseload diminishes over time.

## Discussion

The extent to which surgical treatments are adopted by clinicians must be assessed on a population basis, since studies on clinical or physician-based data are often flawed by bias due to referral patterns or patient selection [21]. Cancer registries can accurately identify cancer cases providing additional information on stage and histology, although the delay in data availability and the coverage of restricted geographical area generally do not allow a timely capture of variability in patterns of care at a regional level. Because all types of breast surgery (including BCS) are performed in hospital settings and not in ambulatory care in Italy, the regional discharge database, which collects data from both public and private structures, ensures a complete coverage and allows proper evaluations of time-trends. The major drawback of this database is that it contains limited data on the stage of disease and comorbidity; improvement of accuracy and completeness of HDR may allow a better evaluation of appropriateness of surgical treatments [22]. As a consequence, the increase in breast conservation may be due both to changing attitudes of patients and clinicians and to the diffusion of screening programs with an increased detection of early-stage cancers. However, this does not hamper the main finding of the present study that the decline of volume-associated differences between structures parallels the growing recourse to breast conservation.

A collaborative study of European tumor registries demonstrated that from 1990 to 1991 only 30.6% of breast cancer patients were treated conservatively in Italy, a proportion far lower than in Northern Europe. Out of the Italian registries, the lowest percentage was reported from Southern Italy [23]. Since the early nineties, the recourse to BCS in some Italian regions was investigated by means of HDR. Overall, these studies were consistent in observing a greater proportion of BCS among younger women, with lower severity of disease, and in those treated in higher-volume hospitals [12-15]; more recent studies reported higher BCS rates than earlier papers, suggesting an increase in breast conservation over time in Italy. The first investigation was performed in the Lombardia Region on 1990–1991 discharges with a rate of breast conservation equal to 43% [12]; a study on 1997 HDR in the Latio Region found 52% conservative procedures [13]; lastly, the BCS reached 67% of total breast cancer surgeries in the Piedmont Region in 2000–2002 [14].

As well as age, stage of disease, and hospital case volume, other factors both at the patient and at the provider level have been reported to influence the choice of breast cancer treatment including socioeconomic status [10], proximity of residence to radiation therapy facilities [24], detection of the malignancy through screening programs [25], academic affiliation and breast surgery experience of individual physicians [26].

Hospital volume is usually defined as the average number of a specific type of surgery in a given hospital over a variable span of years [10]. Our data show two possible intricacies with this approach: variation of hospital volume by calendar year, and time trends in the role played by hospital volume. Modern health care systems are characterized by rapid changes in the profile of providers including structures stop, transfer or begin activity, or sharply modify their activity over time (usually, but not necessarily, with an increase in volume – see Figure 1).

An investigation comparing treatment of early breast carcinoma before and after the introduction of clinical practice guidelines in Australia found an increased proportion of patients undergoing BCS; the analysis by surgeon's caseload showed an increase over time across all classes of activity level except for the least active surgeons [27]. Surgeon's volume is not obtainable from HDR, but is only one facet of the complex association between pattern of care and hospital volume. In our database it was difficult to disentangle separate effects of case-volume, presence of radiation therapy facilities, academic affiliation. Centers with higher volumes may also have a greater capacity to disseminate information on new clinical practices and to assure better access to postoperative therapy.

## Conclusion

The present study delineates an evolving scenario in the Veneto Region, with two coexisting trends: the decline in the proportion of breast cancer patients treated in low-volume hospitals, and the diffusion of breast conserving practices. Larger centers can act as early adopters of new therapeutic strategies and subsequently spread these strategies to other providers, a pattern consistent with the diffusion of innovation theory [4].

## Competing interests

The author(s) declare that they have no competing interests.

## Authors' contributions

UF conceived the study, participated in its design, drafted the manuscript

NA planned, performed and revised the statistical analysis

ES performed the statistical analysis

CV participated in the study design and collected the data

MZ participated in the study design and drafted the manuscript

RR participated in the study design and drafted the manuscript

GR helped to draft and revised the manuscript

PS conceived the study and participated in its design

All authors read and approved the final manuscript

## Pre-publication history

The pre-publication history for this paper can be accessed here:


